# Astaxanthin Treatment Reduced Oxidative Induced Pro-Inflammatory Cytokines Secretion in U937: SHP-1 as a Novel Biological Target

**DOI:** 10.3390/md10040890

**Published:** 2012-04-10

**Authors:** Lorenza Speranza, Mirko Pesce, Antonia Patruno, Sara Franceschelli, Maria Anna de Lutiis, Alfredo Grilli, Mario Felaco

**Affiliations:** Department of Medicine and Science of Aging, University G. D’Annunzio-Chieti, Chieti 66100, Italy; Email: mirkopesce@unich.it (M.P.); antoniapatruno@unich.it (A.P.); s.franceschelli@unich.it (S.F.); m.delutiis@unich.it (M.A.L.); algrilli@unich.it (A.G.); mfelaco@unich.it (M.F.)

**Keywords:** carotenoids, SHP-1 protein, inflammation, astaxanthin

## Abstract

It has been suggested that oxidative stress activates various intracellular signaling pathways leading to secretion of a variety of pro-inflammatory cytokines and chemokines. SHP-1 is a protein tyrosine phosphatase (PTP) which acts as a negative regulator of immune cytokine signaling. However, intracellular hydrogen peroxide (H_2_O_2_), generated endogenously upon stimulation and exogenously from environmental oxidants, has been known to be involved in the process of intracellular signaling through inhibiting various PTPs, including SHP-1. In this study, we investigated the potential role of astaxanthin, an antioxidant marine carotenoid, in re-establishing SHP-1 negative regulation on pro-inflammatory cytokines secretion in U-937 cell line stimulated with oxidative stimulus. ELISA measurement suggested that ASTA treatment (10 µM) reduced pro-inflammatory cytokines secretion (IL-1β, IL-6 and TNF-α) induced through H_2_O_2_, (100 µM). Furthermore, this property is elicited by restoration of basal SHP-1 protein expression level and reduced NF-κB (p65) nuclear expression, as showed by western blotting experiments.

## 1. Introduction

It is known that excess of reactive oxygen species (ROS) is associated to inflammation, growth and vasoconstriction contributing to vascular injury in many cardiovascular diseases, such as hypertension, hyperlipidemia, and diabetes [1,2,3]. Inflammation, a self-defense reaction against various pathogenic stimuli, it may become a harmful self-damaging process, if it transforms to chronic inflammation [4]. Carotenoids are phytochemicals considered beneficial in the prevention of a variety of major diseases [5,6]. The marine carotenoid astaxanthin (ASTA) is naturally found in a wide variety of living organisms, such as microalgae, fungi, and crustaceans [7]. Astaxanthin (3,3-dihydroxy-beta,beta-carotene-4,4-dione) belongs to the xanthophyll subclass of carotenoids. Several studies have demonstrated that ASTA possesses powerful antioxidant properties, both *in vitro* and *in vivo*, especially as an inhibitor of LDL oxidation [8]. Evidence has suggested that the action of carotenoids on immunity and diseases may be mediated, at least in part, by their ability to quench and/or blench ROS [9]. In recent years, a number of studies on astaxanthin have demonstrated its *in vitro* and *in vivo* antioxidant effect, for example, the quenching effect on singlet oxygen, a strong scavenging effect on superoxide, hydrogen peroxide, and hydroxyl radicals and an inhibitory effect on lipid peroxidation. The specific molecular mechanisms of its actions are not yet established [10,11]. Protein tyrosine phosphorylation (PTPs) plays a variety of significant roles in cell signaling transduction, physiological functions, and pathological processes [12,13]. In the PTP family, a subgroup of cytoplasmic PTPs characterized by containing two Src homology 2 (SH2) *N*-terminal domains and a *C*-terminal protein-tyrosine phosphatase domain are referred to as SHP. They are intimately involved in several cellular activities, such as cytoskeletal maintenance, cell division, and cell differentiation [14,15]. Phosphorylation of proteins serves to alter their activity, providing a simple and mostly reversible change in molecular function. Interest in the diverse biology of protein tyrosine phosphatases that are encoded by more than 100 genes in the human genome continues to grow at an accelerated pace [16]. In particular, two cytoplasmic protein tyrosine phosphatases composed of two Src homology 2 (SH2) *N*-terminal domains and a *C*-terminal protein-tyrosine phosphatase domain referred to as SHP-1 and SHP-2 are known to govern a host of cellular functions [17,18].

Most recently, SHP-1 deficiency was found to increase inflammatory gene expression and enhance activation of transcription factor STAT6, STAT1, and NF-κB in PBMC and macrophages of patients with multiple sclerosis [19,20]. Because oxidants are released early in inflammation and have been found to regulate transcription factors, the aim of the present study was to evaluate the *in vitro* effect of carotenoid astaxanthin (ASTA) in inflammation through the evaluation of cytokine release, SHP-1 expression and reactive oxygen species production on U937 cells stimulated with H_2_O_2_.

## 2. Results and Discussion

### 2.1. Cell Viability

It has been well established that oxidative agents, such as H_2_O_2_, can induce cell death [21]. In order to investigate the influence of ASTA on immunitary cell viability, we treated U937 cells with H_2_O_2_, and examined its effects. Since a concentration of 100 µM H_2_O_2_ and an incubation time of 12 h were previously identified as optimal time and concentration (data not shown) for the induction of deleterious effects on U937 cell viability, these conditions were selected for the rest of experiments. The viability of cells exposed to 100 µM H_2_O_2_ for 12 h was 61.5 ± 2.8% *vs.* 96.0 ± 2.8% of the control value, while the viability of cells that were pre-treated for 24 h with ASTA at a concentration of 10 μM prior to 12 h exposure to H_2_O_2_ increased significantly to 78.1 ± 1.9% (*p* < 0.01) (Table 1). These results indicate that the viability of H_2_O_2_-treated cells decreased significantly, but that the ASTA exerted a protective effect against the H_2_O_2_-induced cytotoxicity.

**Table 1:** Effect of astaxanthin (ASTA) upon cell viability evaluated by MTT assay. U937 cells were pre-treated with ASTA (24 h, 10 µM) and followed by adding 100 µM H_2_O_2_. Data are mean ± SD (*n* = 6).

	Before Incubation (%)	After Incubation (%)
CTRL cells	96.9 ± 2.4	96.0 ± 2.8
H_2_O_2_ 100 µM		61.5 ± 2.8 *
H_2_O_2_ 100 µM + ASTA 10 µM		78.1 ± 1.9 ^#^
ASTA 10 µM		97.0 ± 2.2

* *p* < 0.01 *vs.* control cells; ^#^ *p* < 0.01 *vs.* H_2_O_2_ treated cells.

### 2.2. Cytokines Elisa

**Figure 1:**
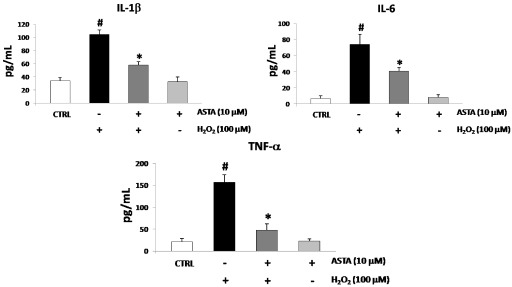
IL-1β, IL-6 and TNF-α levels in U937 cells treated with H_2_O_2_ (100 µM) and treated or non-treated with astaxanthin (ASTA, 10 µM). Cytokines levels resulted augmented after H_2_O_2_ treatment. Co-treatment with ASTA significantly reduced cytokines release, while ASTA alone not affect cytokines released in the culture medium. Values are expressed as pg/mL. Each value represents the mean ± SD of three independent experiments (* *p* < 0.05 *vs.* H_2_O_2_ treated cells, ^#^*p* < 0.05 *vs.* control cells).

Oxidative stress has been shown to promote the production of several cytokines, including pro-inflammatory cytokines IL-1β, IL-6 and TNF-α. U937 cells were treated with either H_2_O_2_ or ASTA to investigate the characteristics of pro-inflammatory cytokines production. The single treatment with H_2_O_2_ (100 µM) was made for 12 h of culture, whereas the single treatment with ASTA (10 µM) was performed during the 24 h culture. In the combined treatment experiment, U937 cells were treated with 10 µM of ASTA for the first 24 h and then with only 100 µM H_2_O_2_ for 12 h. The IL-1β, IL-6 and TNF-α secretion was intensely induced by stimulating the cells with H_2_O_2_ compared with the control, but was significantly lower when pre-incubated for 24 h with ASTA before H_2_O_2_ stimulation (Figure 1).

### 2.3. Effect of H_2_O_2_ and ASTA on the NF-κB Activity in U937 Cells

**Figure 2:**
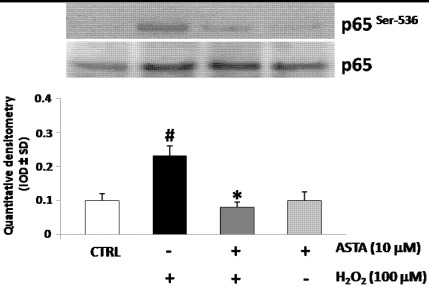
Western blotting analysis of phosphorylated subunit p65 of nuclear factor kappa-light-chain-enhancer of activated B cells (NF-κB) expression in U937 nuclear protein extracts. Cells were pre-treated or not with astaxanthin (ASTA, 10 µM) and following treated with H_2_O_2_. Densitometric analysis is expressed as mean ± SD intensity of optical density (IOD) obtained by three independent experiments (* *p* < 0.05 *vs.* H_2_O_2_ treated cells, ^#^*p* < 0.05 *vs.* control cells).

Reactive Oxygen Species (ROS) may activate nuclear factors, such as NFkB, leading to the production of pro-inflammatory cytokines, which in turn enhance inflammation and, therefore, the generation of other reactive species [22]. In most cases, NF-κB exists in a heterodimeric form composed of p65 (or RelB) and p50. Furthermore, phosphorylation of the p65 subunit at Ser 536 is associated with activation of NF-κB [23,24]. To identify the NF-κB activation and nuclear translocation after H_2_O_2_ stimulation, the phosphorylation levels of p65 subunit were measured by western blot analysis using a phospho-p65-specific Ab anti p-Ser-536 in U937 cells nuclear protein extracts after H_2_O_2_ treatment. Also, to test whether the ASTA affect the H_2_O_2_ induced activation of NF-κB, U937 cells were pre-treated with ASTA and then stimulated with H_2_O_2_ for Western blot analysis using a phospho-p65-specific pAb. Figure 2 showed that nuclear translocation of NF-κB containing the p-p65 subunit was strongly induced H_2_O_2_ treatment, but blocked by ASTA pre-treatment. These data suggest NF-κB involvement in ASTA reduction of pro-inflammatory cytokines secretion induced through H_2_O_2_ treatment.

### 2.4. Effect of H_2_O_2_ and ASTA on SHP-1 Expression and Phosphatase Activity in U937 Cells

**Figure 3:**
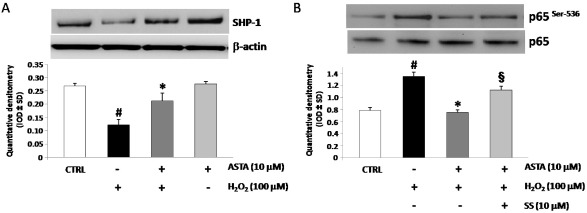
(**A**) Expression of SHP-1 protein in representative Western blot experiments (upper). At the bottom, relative expression of SHP-1 (mean ± SD) in U937 cells pre-treated or not with astaxanthin (ASTA, 10 µM), stimulated with H_2_O_2_ (100 µM) or ASTA aloneand in control cells. Each immunoreactive band was analyzed by densitometry and normalized to β-actin levels. (**B**) At the upper, representative Western blotting experiments of phosphorylated subunit p65 of NF-κB in U937 nuclear protein extracts. Cells were pre-treated with ASTA alone or with ASTA and sodium stibogluconate (SS, 10 µM) and following treated with H_2_O_2_ (bottom). Densitometric analysis is expressed as mean ± SD intensity of optical density (IOD) obtained by three independent experiments (* *p* < 0.05 *vs.* H_2_O_2_ treated cells, ^#^*p* < 0.05 *vs.* control cells, ^§^*p* < 0.05 *vs.* ASTA + H_2_O_2_ treated cells).

Several reports showed inhibitory effect for SHP-1 phosphatase activity on cytokines secretion [25]. In order to investigate a role for SHP-1 in eliciting ASTA effects in U937 cells, western blot using specific anti-SHP-1 pAb experiments were performed. As shown in Figure 3A treatment with H_2_O_2_ strongly down-regulated SHP-1 protein expression. Pre-incubation with ASTA restored SHP-1 basal levels, showing a negative correlation between p65 nuclear translocation and SHP-1 expression. Following this, in order to test whether SHP-1 is involved in the ASTA-mediated inhibition of NF-κB activation, western blot experiments for p-p65 on U937 nuclear extracts protein were performed in the presence of a specific inhibitor of SHP-1 (sodium stibogluconate, SS, 10 µM). Pre-incubation with SS blocked the ASTA-mediated inhibition of NF-κB activity (Figure 3B).

Moreover, pre-incubation with selective SHP-1 inhibitor, blocked ASTA down-regulation of H_2_O_2_ induced cytokines release (Table 2). These data indicate that ASTA blocked the H_2_O_2_ mediated activation of NF-κB and its downstream cytokines secretion through modulating SHP-1 expression.

**Table 2:** IL-1β, IL-6 and TNF-α levels in U937 cells treated with H_2_O_2_ (100 µM) + ASTA (10 µM) and pre-incubated or not with sodium stibogluconate (SS, 10 µM). Cytokines released in the culture medium resulted significantly augmented after pre-treatment with SS. Values are expressed as pg/mL. Each value represents the mean ± SD of three independent experiments.

	IL-1β (pg/mL)	IL-6 (pg/mL)	TNF-α (pg/mL)
CTRL cells	34.2 ± 4.9	7.1 ± 2.9	20.4 ± 8.6
H_2_O_2_ 100 µM	90.5 ± 4.5	66.7 ± 6.0	121.4 ± 11.1
H_2_O_2_ 100 µM + ASTA 10 µM	56.9 ± 8.3	41.5 ± 9.4	48.9 ± 12.2
H_2_O_2_ 100 µM + ASTA 10µM + SS 10 µM	88.9 ± 9.5 *	65.2 ± 4.2 *	122.9 ± 12.5 *

* *p* < 0.05 *vs.* H_2_O_2_ + ASTA treated cells.

## 3. Experimental Section

### 3.1. Cell Culture

U937 mononuclear cells were purchased from American Type Culture Collection (Manassas, VA, USA). The cells were cultured in a 5% CO_2_ atmosphere in RPMI 1640 medium (GIBCO, Invitrogen) containing 10% fetal calf serum, 100 ng/mL streptomycin, 100 U/mL penicillin and 2 mM L-glutamine. Cells derived from the same freeze-down batch were thawed, grown and seeded (at 2 × 10^5^ cells per well) onto six-well tissue culture plates and cultured in medium with and without 10 µM astaxanthin (ASTA), with and without sodium stigluconate (SS) and treated with H_2_O_2_ (100 µM). ASTA was dissolved in dimethyl sulfoxide (DMSO) and diluted with the medium. The final concentration of DMSO in the medium was 0.5%. Control groups not contained ASTA and/or H_2_O_2_. Cell viability was determined by trypan blue dye exclusion and MTT assay (Biotium, Hayward, CA, USA).

### 3.2. Reagents

The primary antibodies used for Western blotting were rabbit anti-SHP-1, anti NF-κB and anti-p-NF-κB (p65) (Santa Cruz Biotechnology, Santa Cruz, CA, USA) and mouse anti-β-actin (A5441; Sigma-Aldrich, St. Louis, MO, USA). Astaxanthin was obtained by Sigma-Aldrich. Sodium stibogluconate was purchased from Merck (Darmstadt, Germany).

### 3.3. Cytokines ELISA

U937 cells were pretreated with ASTA for 24 h. After pre-incubation with ASTA, H_2_O_2_ was added to the wells. In other experiment pre-incubation was performed with ASTA and SS. The supernatants were collected and assayed using the Searchligth Elisa kit according to the manufacturer’s instructions (Thermo Fisher Scientific, Rockford, IL, USA.).

### 3.4. Western Blotting

U937 cells were washed once in cold phosphate-buffered saline (PBS; 0.5 mol/L sodium phosphate, pH 7.5) and harvested by gentle scraping, and used to prepare total protein or nuclear extracts. Total protein extracts were prepared by treating cells with lysis buffer [50 mmol/L Tris–HCl pH 7.5, 0.4% Nonidet P-40 (NP-40), 120 mmol/L NaCl, 1.5 mmol/L MgCl_2_, 2 mmol/L phenylmethylsulphonyl fluoride (PMSF), 1 µg/mL leupeptin, 3 mmol/L NaF and 1 mmol/L dithiothreitol] for 30 min at 4 °C. Nuclear extracts were prepared according to Osborn *et al.* [24]. Cells were pelleted, frozen in dry ice⁄ethanol, resuspended in 75 µL of Buffer A (10 mmol/L HEPES pH 7.9, 10 mmol/L KCl, 0.5 mmol/L EDTA, 1.5 mmol/L MgCl_2_, 0.2% NP-40 and 0.5 mmol/L PMSF) and placed on ice for 10 min to allow lysis. Nuclei were pelleted by centrifugation at 3500 g for 10 min at 4 °C, resuspended in 1 mL of Buffer B (20 mmol/L HEPES pH 7.9, 400 mmol/L NaCl, 1.5 mmol/L MgCl_2_, 0.5 mmol/L EDTA, 25% glycerol and 0.5 mmol/L PMSF) and placed on a rocking platform for 30 min at 4 °C. The nuclear lysates were then clarified by centrifugation at 14,000 g for 20 min at 4 °C and the supernatants (nuclear extracts) collected. The protein concentrations of the extracts were determined using the Bradford method (Bio-Rad protein assay, Hercules, CA, USA). For Western blot analysis, 50 µg of protein per lane was separated on a 4–12% NuPAGE gradient gel (Gibco Invitrogen), electro-transferred on to a nitrocellulose membrane and blocked with 10% skimmed milk in PBS containing 0.1% Tween-20. Blots were probed and incubated overnight at 4 °C with the rabbit polyclonal IgG anti-SHP-1, the rabbit polyclonal IgG anti-pNF-κB and NF-κB (p65) all at 0.2 µg/mL in Tris-buffered saline (TBS)/0.1% Tween-20. A rabbit antihuman monoclonal antibody recognizing the human β-actin was used as control in all experiments. Blots were then washed and incubated for 1 h with goat antirabbit–horseradish peroxidase (Pierce Biotechnology, Rockford, IL, USA) diluted 1:10,000 in TBS/0.1% Tween-20. Immunoblot signals were developed using the Super Signal Ultra chemiluminescence detection reagents (Pierce Biotechnology). The blot images were analyzed with a gel analysis software package (Gel Doc 1000; Bio-Rad, Milan, Italy). Data are expressed as mean ± SD intensity of optical density.

### 3.5. Statistical Analysis

The results are expressed as mean ± SD. Statistical analysis was performed using analysis of variance (ANOVA). The probability of null hypothesis of <5% (*p* < 0.05) was considered statistically significant. The comparison H_2_O_2_* vs.* H_2_O_2_ + ASTA and H_2_O_2_ + ASTA *vs.* H_2_O_2_ + ASTA + SS was performed using post hoc test with the alpha level at 0.05.

## 4. Conclusions

It is well recognized that persistent inflammation contributes to the pathogenesis of many diseases, including cancer, heart disease and atherosclerosis [25,26,27]. The manipulation of the course and intensity of an inflammatory process may occur not only by using agents that inhibit activated “pro-inflammatory pathways”, but also through an approach to activate the natural anti-inflammatory processes, therefore it is essential to identify the metabolic pathways, sensitive to excessive tissue damage, which contribute to the resolution of inflammation. The development of new anti-inflammatory drugs reflects the need to prevent excessive tissue damage that can be established as a result of the persistence of inflammation and at the same time, the need to better understand the processes that contribute to the control and resolution of inflammation. The main properties of astaxanthin inactivate harmful free radicals that are the basis of an inflammatory process. In the immune system, SHP-1 plays critical roles in regulation of many receptor-mediated signaling cascades, and SHP-1 deficiency in mice causes spontaneous inflammation and autoimmunity [28,29]. For the first time our results show that astaxanthin most likely inhibits ROS-induced production of NF-κB transcription factor, which in turn effectively inhibits the production of inflammatory cytokines, through a restoration of physiological levels of SHP-1. Astaxanthin has potential and promising applications in human health because it represents the new frontier of therapy against free radicals [30]. Our future aim will be to demonstrate that the ASTA through the positive regulation of SHP-1 could represent a new therapeutic approach in relation to common inflammatory diseases, even for those affected with allergies cannot benefit from the use of Non-steroidal anti-inflammatory drugs (NSAIDs). In addition, in relation to these findings our goal is to propose the *in vitro* and *in vivo* evaluation of ASTA as a positive modulating factor in diseases such as rheumatoid arthritis, where persistent inflammation leads to constant and harmful use of anti-inflammatory molecules (NSAIDs and glucocorticoids).
